# Different Formulations and Concentrations of Sweet Chestnut Tannins Differentially Modulate Gut Microbiota in Broiler Chickens

**DOI:** 10.3390/ani16213514

**Published:** 2026-11-06

**Authors:** Isidora Prošić, Maksimiljan Brus, Nikola Delić, Nemanja Zdravković, Andrea Radalj, Tamaš Petrović, Vladimir Gajdov, Jakov Nišavić, Aleksandar Nikšić, Mila Milinković, Dragan Damjanović, Milica Čilerdžić, Dejan Krnjaić

**Affiliations:** 1 Department of Microbiology and Immunology, Faculty of Veterinary Medicine, University of Belgrade, Bul. Oslobodjenja 18, 11000 Belgrade, Serbia; andrea.zoric@vet.bg.ac.rs (A.R.); jakovmoni@vet.bg.ac.rs (J.N.); aleksandar.niksic@vet.bg.ac.rs (A.N.); milamilinkovic.mm@gmail.com (M.M.); dejan.krnjaic@vet.bg.ac.rs (D.K.); 2 Department of Livestock Breeding and Nutrition, Faculty of Agriculture and Life Sciences, University of Maribor, Slomškov trg 15, 2000 Maribor, Slovenia; maksimiljan.brus@um.si; 3 Institute of Animal Husbandry, Zemun Autoput Beograd-Zagreb 16, 11000 Belgrade, Serbia; delicnikola68@yahoo.com; 4 Department of Bacteriology and Parasitology, Scientific Veterinary Institute of Serbia, Janisa Janulisa 14, 11000 Belgrade, Serbia; nemanja.zdravkovich@gmail.com; 5 Department of Virology, Scientific Veterinary Institute “Novi Sad”, Rumenacki put 20, 21000 Novi Sad, Serbia; tomy@niv.ns.ac.rs (T.P.); vladimir.g@niv.ns.ac.rs (V.G.); 6 Korvet, Zaplanjska 86a, 11000 Belgrade, Serbia; dragan.damnjanovic@korvetteam.rs; 7 Aviaid Consulting, Slikara Milovanovića 3, 37000 Kruševac, Serbia; milicacilerdzic5@gmail.com

**Keywords:** antibiotic alternatives, broilers, caecum, hydrolysable tannins, ileum, microbial diversity, microencapsulation, phytogenic feed additives, 16S rRNA gene sequencing

## Abstract

Sweet chestnut tannins (SCT) are used as phytogenic feed additives in poultry, but the effects of different formulations and concentrations on intestinal microbiota remain poorly understood. This study investigated powdered, soy protein-bound, microencapsulated and orthophosphoric acid-bound SCT at different dietary concentrations in sixty Ross 308 broilers, with six birds per group, including an unsupplemented control. Ileal and caecal microbiota were characterized using 16S rRNA gene sequencing. In the ileum, richness, phylogenetic diversity and beta diversity differed among treatments, whereas Shannon diversity and evenness remained unchanged. *Lactobacillaceae* remained dominant, and no significant family- or genus-level differences relative to the control remained after multiple-testing correction. In the caecum, all assessed alpha- and beta-diversity metrics differed among treatments, with numerous treatment-specific taxonomic changes. Several treatments increased the Firmicutes/Bacteroidota ratio, while *Muribaculaceae* decreased across most supplemented groups. Soy protein-bound SCT at 1000 mg/kg increased *Parabacteroides* and *Odoribacter*, whereas microencapsulated SCT at 1000 mg/kg increased *Romboutsia* and *Peptostreptococcaceae*. The genus *Fournierella* increased with soy protein-bound SCT at 2000 mg/kg. Responses were not consistently dose-dependent. These findings indicate that SCT-associated microbial changes vary with intestinal compartment, formulation and concentration, with broader responses detected in the caecum. Their implications for bird health and performance require further investigation.

## Simple Summary

Sweet chestnut tannins are plant compounds used in poultry feed to support intestinal health. However, how different formulations and dietary concentrations influence intestinal bacteria remains largely unclear. This study compared four tannin formulations at different dietary concentrations in sixty broiler chickens. Bacterial communities were examined in the ileum, a major site of nutrient absorption, and the caeca, where bacteria ferment undigested food. The main bacterial groups in the ileum remained largely stable, whereas more extensive changes in bacterial diversity and composition occurred in the caeca. Soy protein-bound tannins at 1000 milligrams per kilogram of feed increased the genera *Parabacteroides* and *Odoribacter*, which are associated with the fermentation of dietary carbohydrates and the production of compounds that may support intestinal health. Microencapsulated tannins at the same concentration increased the genus *Romboutsia*, which is also involved in carbohydrate fermentation. Higher tannin concentrations did not consistently produce stronger responses, highlighting the importance of selecting an appropriate concentration. These findings can guide the development of tannin-based feeding strategies for poultry production. However, measurements of bacterial products, bird health and growth are needed before these changes can be considered beneficial.

## 1. Introduction

Poultry meat is a major source of animal protein globally owing to its relatively low production costs, high nutritional value and broad consumer acceptance [1,2]. According to OECD and FAO projections, it will account for approximately 45% of meat-derived protein by 2034 [2], highlighting the importance of maintaining poultry productivity and health.

The poultry gut microbiota contributes to digestion, nutrient utilization, immune development, intestinal barrier integrity and resistance to pathogen colonization [3,4]. Its composition varies along the digestive tract: the ileum supports nutrient digestion and absorption and harbors relatively low microbial diversity, whereas the caeca contain dense, diverse communities that ferment undigested substrates and produce short-chain fatty acids (SCFAs) [4]. Nutritional strategies targeting these communities have therefore attracted increasing interest in poultry production.

For decades, antibiotic growth promoters were widely used to improve growth performance and maintain intestinal health in poultry. However, concerns regarding the contribution of antimicrobial use to the development and spread of antimicrobial resistance have led to restrictions on antibiotic growth promoters and stimulated the search for alternative feed additives capable of supporting animal health and productivity [5,6]. Among the proposed alternatives, phytogenic feed additives have emerged as promising alternatives owing to their antimicrobial, antioxidant, anti-inflammatory, and immunomodulatory properties [7,8,9].

Tannins are naturally occurring polyphenolic compounds widely distributed throughout the plant kingdom, where they serve as protective secondary metabolites involved in plant defense and are broadly classified into hydrolysable and condensed tannins [10,11]. Although traditionally regarded as antinutritional factors in poultry nutrition due to their ability to bind proteins and other macromolecules [11,12], more recent studies have demonstrated that selected tannin preparations can exert beneficial effects on poultry health and productive performance when administered at appropriate concentrations [12]. In particular, hydrolysable tannins derived from sweet chestnut (*Castanea sativa* Mill.) have been associated with improvements in gut health and productive performance in broiler chickens [13,14,15] and have also demonstrated significant antimicrobial activity against pathogenic bacteria, including avian pathogenic *Escherichia coli* [9]. Dietary chestnut- and quebracho-derived tannins can alter caecal microbial composition and diversity through selective effects on bacterial taxa [10,12]. However, findings are inconsistent. Daghio et al. observed no significant changes in caecal alpha or beta diversity with chestnut tannin extract, alone or combined with short- and medium-chain fatty acids [16]. Formulation may influence tannin bioavailability and intestinal delivery. Encapsulation, for example, can reduce interactions with dietary proteins and increase delivery to the distal intestine [17].

Despite increasing evidence supporting the beneficial effects of tannins in poultry production, relatively little is known about how different tannin formulations and concentrations influence the structure and composition of the gut microbiota [17,18]. To address this gap, we compared four sweet chestnut tannin formulations at inclusion levels selected based on previous research conducted by the manufacturer. The main comparisons were treatments versus the control, concentrations within formulations, and formulations at matched inclusion levels. We expected these comparisons to help identify candidate formulations and concentrations for subsequent studies of microbial metabolites and bird performance. The novelty of this study lies in examining these treatments in both ileal and caecal microbiota using 16S rRNA gene amplicon sequencing to test the hypothesis that microbial responses differ according to formulation, dietary concentration and intestinal compartment.

## 2. Materials and Methods

### 2.1. Experimental Design and Sample Collection

The study was conducted under experimental conditions at the Institute for Animal Husbandry, Belgrade, Serbia. All animal procedures were approved by the Veterinary Directorate of the Ministry of Agriculture, Forestry and Water Management of the Republic of Serbia (Approval No. 323-07-08802/2022-05). A total of 250 female Ross 308 broiler chickens were allocated to ten dietary treatment groups, with 25 birds per treatment. The groups comprised an unsupplemented control and nine groups receiving different formulations and concentrations of sweet chestnut tannins. Each treatment comprised six separate floor pens, five housing four birds each and one housing five birds. Each pen had a floor area of 1 m^2^, resulting in a stocking density of four or five birds/m^2^. Pens were labelled according to a predetermined treatment sequence before bird placement. Birds were then allocated to the designated pens by drawing individuals at random from the initial flock. Birds were housed under standard and controlled husbandry conditions, with an automated ventilation system and an 18-h light cycle, and were fed commercial diets formulated according to the breeder’s recommendations, with ad libitum access to feed and water throughout the 42-day experimental period. All birds were vaccinated on day 1 against Newcastle disease using Nobilis^®^ ND Clone 30 (Nobilis® ND Clone 30 (MSD Animal Health, Boxmeer, the Netherlands). No medications were administered during the experimental period. On day 42, six birds from each treatment group were selected for microbiota analysis, with one bird sampled from each of the six distinct pens per treatment (60 birds in total). Within each pen, the first bird caught was selected for sampling. The pen was considered the experimental unit, with one sampled bird representing each pen. The experimental design is presented in Table 1.

The compositions and calculated tannin contents of the formulations are presented in Table 2. TSP was prepared by thermally treating raw soybeans and subsequently mixing the resulting soybean protein material with sweet chestnut tannin extract at a 1:1 ratio (*w*/*w*). TMC contained extract microencapsulated in palm oil, while TPA was modified by acid hydrolysis with orthophosphoric acid. Dietary inclusion levels refer to the complete commercial preparations, not active tannins. Birds received starter (days 1–14), grower (days 15–28) and finisher (days 29–42) diets. Their ingredient and calculated nutrient compositions are presented in Table 3.

On day 42, birds were humanely euthanized by cervical dislocation. Sampling began in the morning, with ten operators working in parallel so that birds from different treatment groups were sampled at approximately the same time. Approximately 1.5 g of digesta was collected from the distal ileum and from the pooled contents of both caeca per bird. Samples were individually labelled, immediately placed on ice and transported to the laboratory, remaining on ice for 0.5–3 h before DNA extraction.

### 2.2. DNA Extraction

Digesta samples were not frozen. DNA was extracted from all samples in a single batch on the collection day, and the extracts were stored at −20 °C for 2 weeks. Sampling day and extraction batch were therefore common to all treatments. Total genomic DNA was extracted from ileal and caecal samples using the EURx Stool DNA Purification Kit (EURx, Gdańsk, Poland) according to the manufacturer’s instructions. DNA concentration and purity were assessed spectrophotometrically using a BioSpec-nano UV–VIS spectrophotometer (Shimadzu, Japan). Extracted DNA samples were submitted to Novogene Co., Ltd. (Beijing, China) for commercial 16S rRNA gene amplicon library preparation and high-throughput sequencing.

### 2.3. 16S rRNA Gene Amplification and Illumina MiSeq Sequencing

The V3–V4 hypervariable region of the bacterial 16S rRNA gene was amplified using the universal primers 341F (CCTAYGGGRBGCASCAG) and 806R (GGACTACNNGGGTATCTAAT). Amplicon libraries were prepared by Novogene Co., Ltd. (Beijing, China) according to standard Illumina protocols and sequenced on an Illumina MiSeq platform using 2 × 250 bp paired-end library preparation.

### 2.4. Bioinformatic and Statistical Analysis

Raw paired-end Illumina MiSeq reads were processed using QIIME 2 v2026.4 [19]. Sample metadata and sequencing reads were imported into QIIME 2, and sequence quality was assessed using the demux summarize plugin. Because read merging performance differed between intestinal compartments, separate processing workflows were applied to caecal and ileal datasets. For the caecal dataset, paired-end reads were denoised using the DADA2 plugin implemented in QIIME 2. Reads were quality-filtered, denoised, merged, and screened for chimeras using the DADA2 denoise-paired workflow. Representative sequences and amplicon sequence variants (ASVs) abundance tables were generated for downstream analyses. For the ileal dataset, initial processing with DADA2 resulted in substantial sequence loss during paired-end merging due to insufficient overlap between forward and reverse reads after quality filtering. Therefore, reads were merged using VSEARCH and subsequently denoised using Deblur, implemented in QIIME 2. Representative sequences from both datasets were aligned using MAFFT, and phylogenetic trees were constructed using FastTree through the QIIME 2 align-to-tree-mafft-fasttree pipeline.

Taxonomic classification was performed using the SILVA 138.2 SSURef NR99 weighted classifier trained on full-length animal distal gut sequences [20]. Taxonomic names are reported according to the nomenclature provided by the SILVA 138.2 reference database used for taxonomic classification. Taxonomic assignments were generated for all ASVs and subsequently collapsed to phylum, family, and genus levels for community composition analyses. Alpha diversity metrics (observed ASVs, Shannon diversity index, Faith’s phylogenetic diversity, and Pielou’s evenness) were calculated after rarefaction to a standardized sequencing depth (29,455 for the caecal dataset, 15,086 for the ileal dataset) selected based on sequencing depth distributions and rarefaction curve inspection. For diversity analyses, feature tables were rarefied to 15,086 sequences per sample for the ileal dataset and 29,455 sequences per sample for the caecal dataset. These depths were selected based on sequencing depth distributions and inspection of rarefaction curves (Supplementary Figure S4), allowing all samples to be retained for diversity analyses. Rarefaction standardized sequencing depth within each intestinal compartment, reducing the influence of unequal read counts on diversity comparisons. However, subsampling may result in the loss of low-abundance taxa and influence richness-sensitive diversity metrics, particularly at lower rarefaction depths.

Statistical differences in alpha diversity among experimental groups were assessed using the QIIME 2 alpha-group-significance plugin. Beta diversity was evaluated using Bray–Curtis, Jaccard, weighted UniFrac, and unweighted UniFrac distance metrics. Differences in community composition were tested using PERMANOVA implemented in QIIME 2. PERMANOVA assumes exchangeability of observations under the permutation scheme, and significant results may be influenced by differences in within-group dispersion. Pairwise comparisons between inclusion levels within each formulation were interpreted as exploratory analyses of concentration-associated differences. For downstream analyses, feature tables and taxonomic assignments were exported from QIIME 2 and further processed using R v. 4.6.1. Unless otherwise stated, data are presented as mean ± standard deviation (SD). Differential abundance analyses were conducted using ANCOM-BC2 v2.0.2 [21]. Genus- and family-level abundance data were imported into R using the phyloseq package. Global, pairwise, and Dunnett-type comparisons were performed. *p*-values were adjusted using the Holm method, and taxa were considered significantly different when adjusted *p*-values were <0.05 and passed ANCOM-BC2 sensitivity analysis. Although ANCOM-BC2 accounts for compositionality and sampling biases, results depend on model assumptions and may be less reliable for low-abundance taxa.

## 3. Results

### 3.1. Ileal Microbiota

#### 3.1.1. Sequencing Statistics

Following paired-end read merging, a total of 5,118,424 merged sequences were obtained from 60 ileal samples, corresponding to an average of 85,307 sequences per sample (range: 43,689–107,404). The merged sequences exhibited a highly uniform length distribution, with 90.61% measuring 429 bp. Quality score profiles demonstrated consistently high base-calling accuracy throughout the reads, with median Phred scores remaining above Q30 across most positions. No samples were excluded for downstream analyses. Following Deblur denoising, 15,298–42,696 reads per sample were retained, with 15,086–42,234 reads remaining after reference matching. Read retention after Deblur denoising ranged from 30.00% to 45.55% of the merged input sequences, while retention after reference matching ranged from 28.17% to 45.05%. Following Deblur denoising, all 60 samples were retained for downstream analysis. The final feature table contained 1081 unique ASVs and 1,831,865 total observations. Individual ASV frequencies ranged from 10 to 608,035 reads, whereas the median ASV abundance was 40 reads. Detailed sequencing and Deblur processing statistics for individual ileal samples are provided in Supplementary Table S1.

#### 3.1.2. Alpha Diversity

Observed ASV richness differed significantly among groups (H = 20.80, *p* = 0.0135) and Faith’s phylogenetic diversity differed significantly across groups (H = 20.99, *p* = 0.0127). Shannon diversity did not differ significantly among groups (H = 10.80, *p* = 0.2899). Pielou’s evenness was not significantly affected (H = 7.45, *p* = 0.5908). Although overall differences were detected for Observed Features and Faith’s phylogenetic diversity, no pairwise comparisons remained significant after false discovery rate correction (q > 0.05) (Supplementary Figure S1).

#### 3.1.3. Beta Diversity

**Figure 1:**
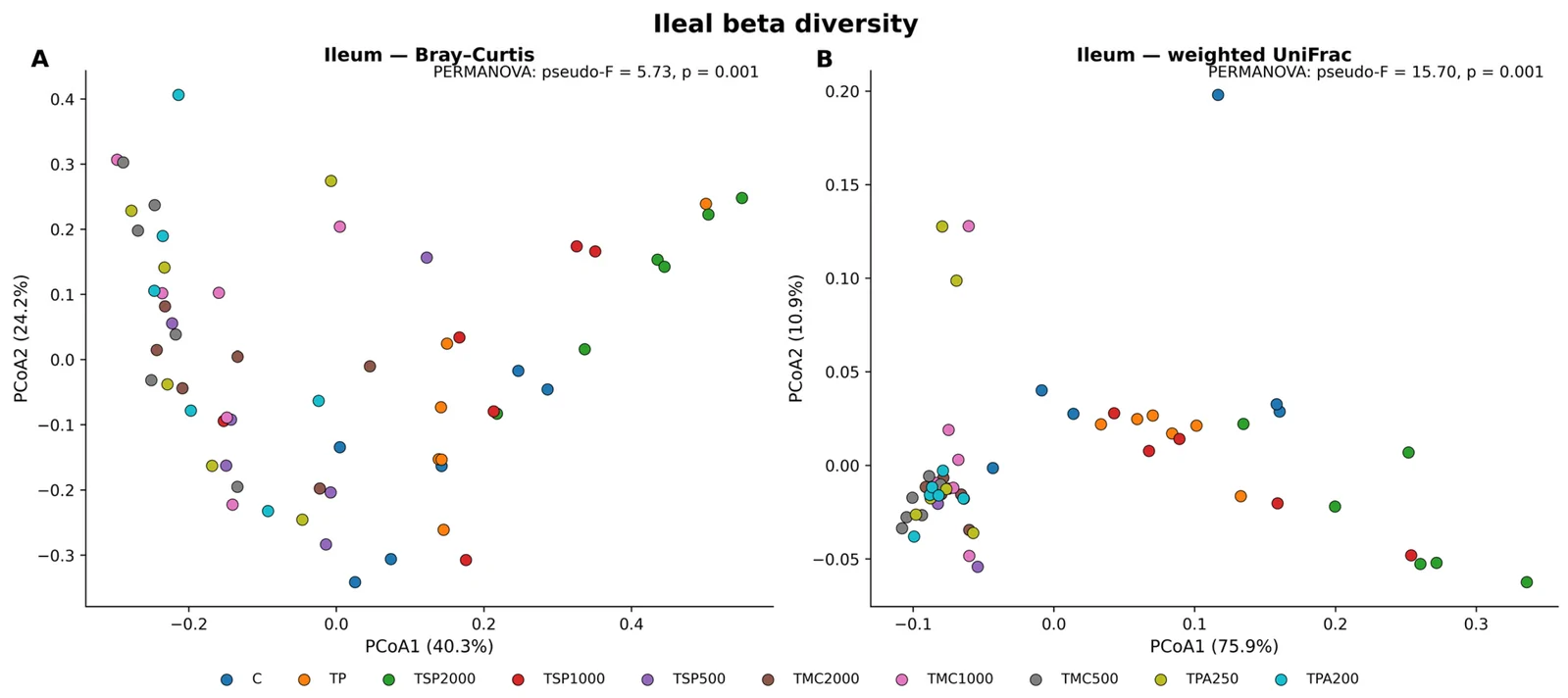
Principal coordinate analysis of ileal microbiota based on (**A**) Bray–Curtis and (**B**) weighted UniFrac distances. Each point represents one bird, and colors indicate dietary treatment groups.

PERMANOVA analyses demonstrated significant differences in community composition among treatment groups for all distance metrics, including Bray–Curtis (pseudo-F = 5.73, *p* = 0.001), Jaccard (pseudo-F = 2.92, *p* = 0.001), unweighted UniFrac (pseudo-F = 3.53, *p* = 0.001) and weighted UniFrac (pseudo-F = 15.70, *p* = 0.001) (Figure 1 and Supplementary Figure S2). Pairwise comparisons showed that TP did not differ significantly from the control for any of the four metrics. TSP1000 differed significantly from the control based on Jaccard and unweighted UniFrac distances, whereas TSP2000 differed significantly based on Bray–Curtis and weighted UniFrac distances. All remaining treatment groups differed significantly from the control across all four metrics. Exploratory within-formulation comparisons identified differences between all TSP inclusion levels, although significance depended on the distance metric. TMC500 differed from TMC2000 across all four metrics and from TMC1000 for Jaccard and both UniFrac metrics. No significant differences were detected between TMC1000 and TMC2000 or between TPA200 and TPA250. Complete pairwise PERMANOVA results are provided in Supplementary Table S2.

#### 3.1.4. Taxonomic Composition of the Ileal Microbiota

**Figure 2:**
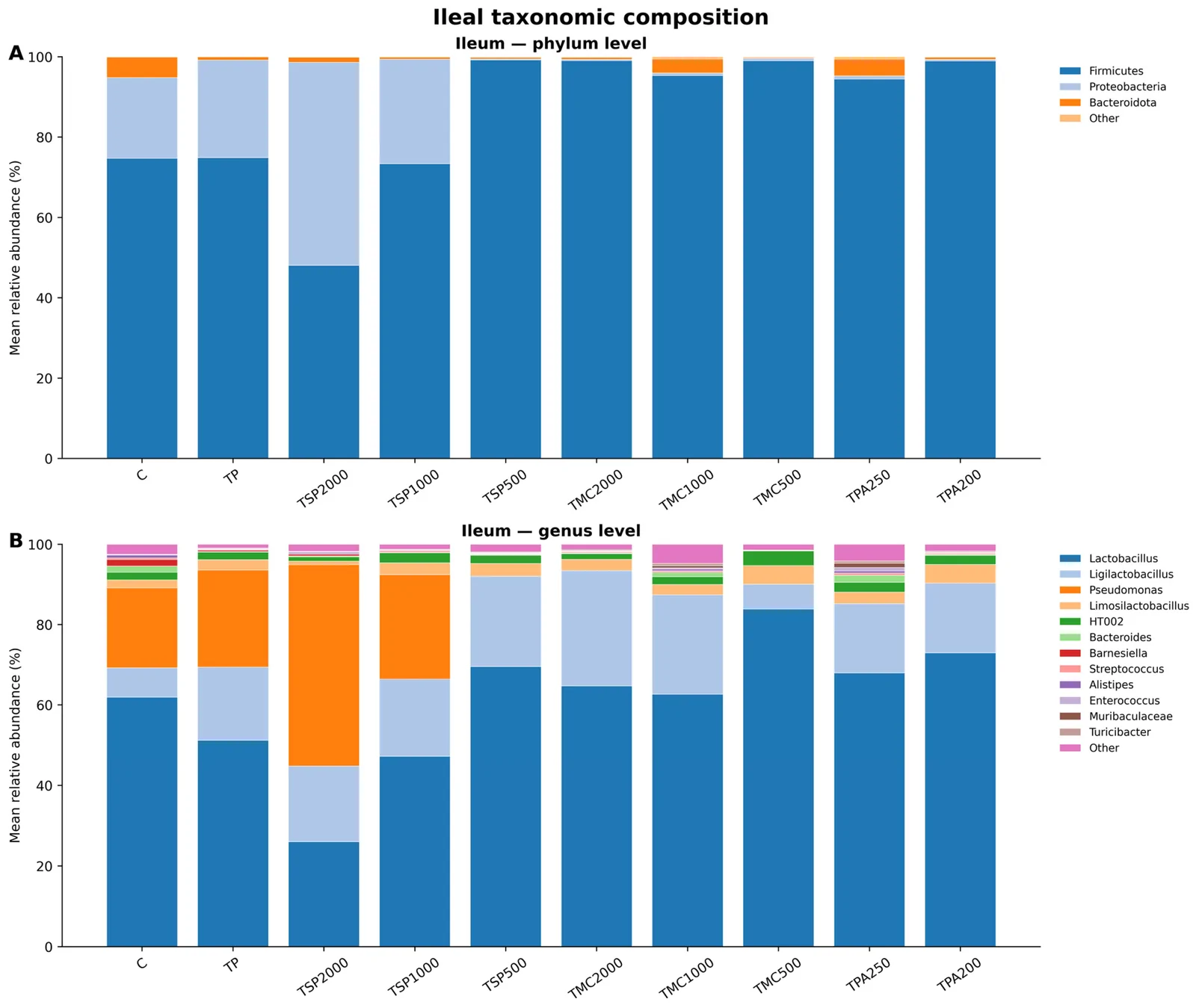
Taxonomic composition of the ileal microbiota at the (**A**) phylum and (**B**) genus levels. Bars show mean relative abundances within each dietary treatment group. HT002 is an unclassified or candidate bacterial genus belonging to the family *Lactobacillaceae*. Less abundant taxa are grouped as “Other”.

At the phylum level, the ileal microbiota was dominated by Firmicutes (85.7%), followed by Proteobacteria (12.5%) and Bacteroidota (1.7%), which together accounted for more than 99% of all sequences. The remaining phyla, including Actinobacteriota, Desulfobacterota, Spirochaetota, Deferribacterota and Verrucomicrobiota, each represented less than 0.1% of the total abundance. Among the 64 identified bacterial families, *Lactobacillaceae* predominated (84.0%) and was detected in all samples, whereas *Pseudomonadaceae* represented the second most abundant family (12.1%). The remaining families occurred at comparatively low abundances. A total of 119 genera were identified across the ileal samples. The community was dominated by *Lactobacillus* (61.3%), *Ligilactobacillus* (17.8%), *Pseudomonas* (12.1%), *Limosilactobacillus* (2.8%) and HT002 (an unclassified or candidate bacterial genus belonging to the family *Lactobacillaceae*) (2.0%). The genera *Lactobacillus*, *Ligilactobacillus*, *Limosilactobacillus* and HT002 accounted for approximately 83.9% of the total community. All remaining genera occurred at relatively low abundances and are presented in Figure 2 and Supplementary Figure S3.

#### 3.1.5. Differential Abundance Analysis of the Ileal Microbiota

Family-level ANCOM-BC2 analysis identified four families with significant global variation among treatment groups: *Aerococcaceae* (W = 7.18, q = 3.80 × 10^−5^), *Staphylococcaceae* (W = 6.21, q = 9.20 × 10^−5^), *Bacillaceae* (W = 4.25, q = 0.0038), and *Streptococcaceae* (W = 3.67, q = 0.0082). *Lactobacillaceae* did not differ significantly among groups. However, despite significant global effects, neither comparisons with the control nor pairwise comparisons among treatment groups remained significant after multiple-testing correction.

At the genus level, six taxa exhibited significant global variation: *Ligilactobacillus* (W = 21.22, q = 7.66 × 10^−13^), *Lactococcus* (W = 12.87, q = 6.46 × 10^−9^), *Staphylococcus* (W = 9.90, q = 5.44 × 10^−7^), *Bacillus* (W = 5.77, q = 3.86 × 10^−4^), *Limosilactobacillus* (W = 4.78, q = 0.0018), and *Aerococcus* (W = 3.75, q = 0.0227). Nevertheless, no genus-level comparisons with the control or between individual treatment groups were significant after correction. Thus, the significant global findings did not translate into statistically significant differences in individual group comparisons. Complete results are provided in Supplementary Table S3.

### 3.2. Caecal Microbiota

#### 3.2.1. Sequencing Statistics

Amplicon sequencing generated 6,392,977 paired-end reads from 60 caecal samples, corresponding to an average of 106,550 reads per sample (range: 89,893–119,622). Sequence quality remained high throughout both forward and reverse reads, with median Phred quality scores exceeding Q30 across the majority of bases. All reads were trimmed to 220 bp prior to denoising. Following DADA2 processing, 76.8–91.0% of reads were retained, and paired-end read merging was successful for most samples, with merge rates generally ranging from 68% to 76% (Supplementary Table S4). Two samples from the TPA200 group exhibited lower merging efficiencies. They retained 41,450 and 29,455 high-quality sequences, respectively, and were therefore included in downstream analyses. Final non-chimeric read retention ranged from 24.7% to 72.9% of input reads. The final feature table contained 4639 ASVs and 4,233,758 non-chimeric reads distributed across all samples. Sequencing depth after denoising remained high, with a mean of 70,563 reads per sample (range: 29,455–85,447). Rarefaction analysis indicated that sequencing depth was sufficient to capture the majority of bacterial diversity in both ileal and caecal samples (Supplementary Figure S4). No samples were excluded from further analyses.

#### 3.2.2. Alpha Diversity

**Figure 3:**
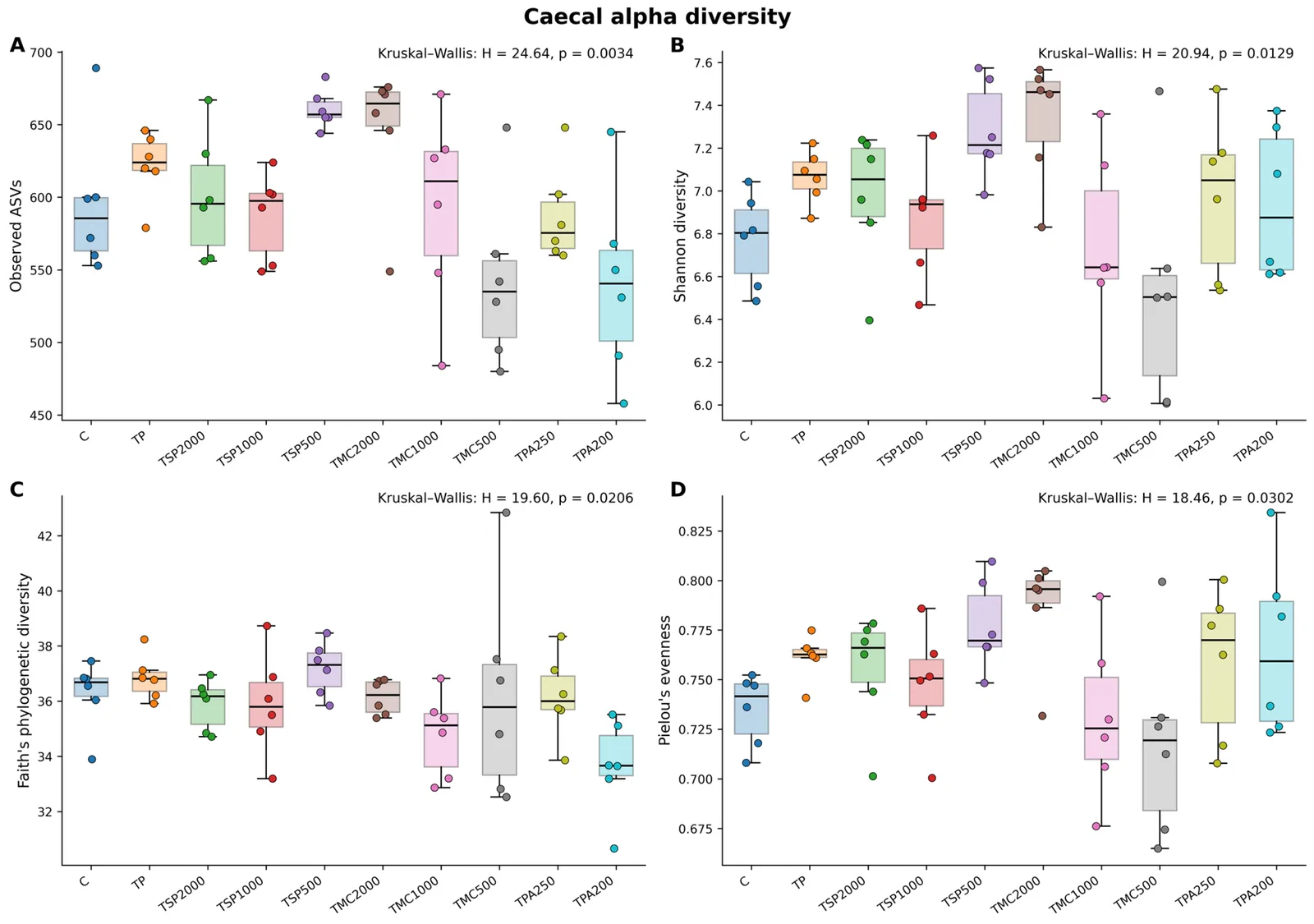
Alpha diversity of the caecal microbiota across dietary treatment groups: (**A**) Observed ASVs, (**B**) Shannon diversity, (**C**) Faith’s phylogenetic diversity, and (**D**) Pielou’s evenness. Boxplots show the median (horizontal line), interquartile range (box, 25th–75th percentiles), and whiskers extending to 1.5 times the interquartile range. Individual points represent biological replicates (*n* = 6 per group). Kruskal–Wallis test statistics (H) and *p*-values are indicated in each panel.

All four alpha-diversity metrics differed significantly among treatment groups, including observed ASV richness (H = 24.64, *p* = 0.0034), Shannon diversity (H = 20.94, *p* = 0.0129), Faith’s phylogenetic diversity (H = 19.60, *p* = 0.0206) and Pielou’s evenness (H = 18.46, *p* = 0.0302) (Figure 3).

#### 3.2.3. Beta Diversity

**Figure 4:**
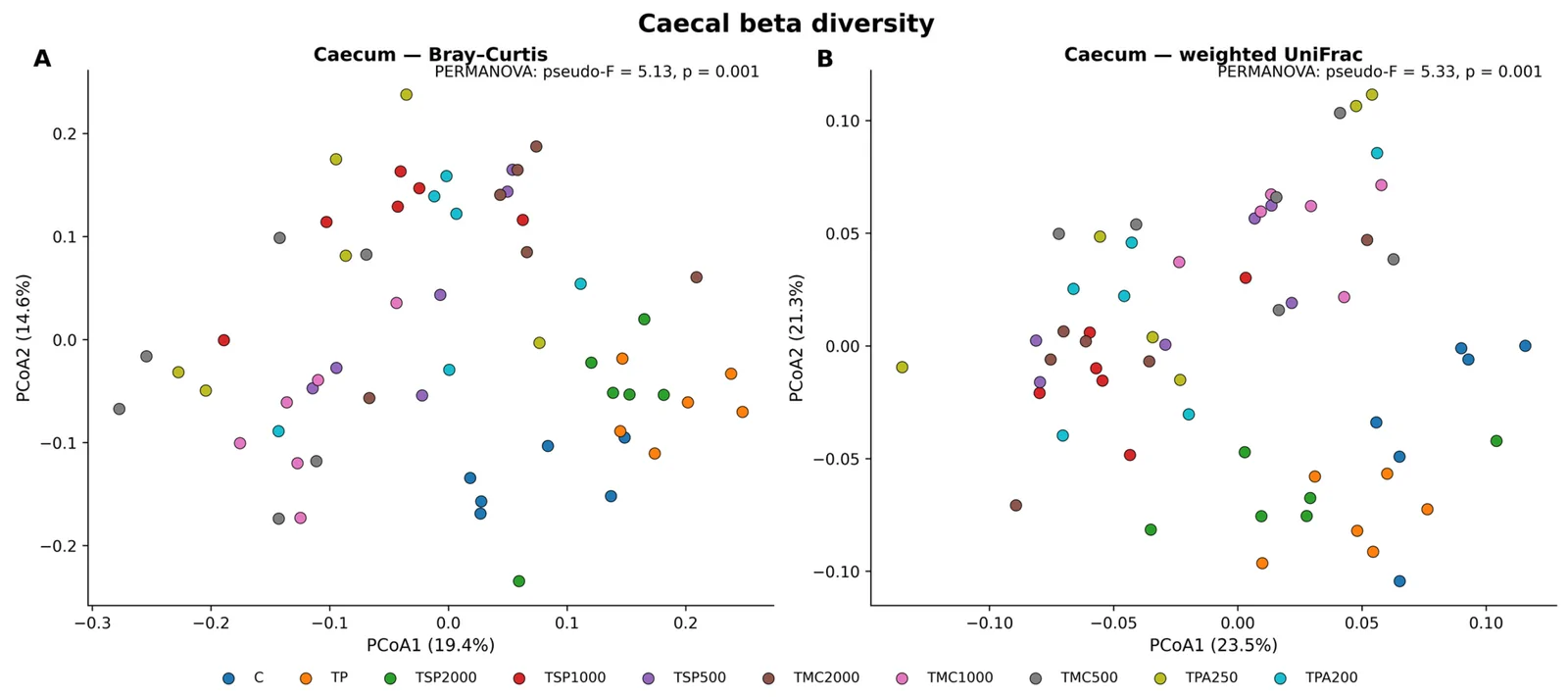
Caecal beta diversity. Principal coordinate analysis based on (**A**) Bray–Curtis and (**B**) weighted UniFrac distances. Each point represents one bird, and colors indicate dietary treatment groups.

PERMANOVA analyses demonstrated significant treatment-associated differences in caecal microbial community composition across all four distance metrics (Bray–Curtis: pseudo-F = 5.13, *p* = 0.001; Jaccard: pseudo-F = 2.31, *p* = 0.001; unweighted UniFrac: pseudo-F = 1.94, *p* = 0.001; weighted UniFrac: pseudo-F = 5.33, *p* = 0.001) (Figure 4 and Supplementary Figure S2). Pairwise comparisons revealed that all nine tannin-treated groups differed significantly from the control across all four distance metrics (FDR-adjusted q < 0.05). The magnitude of these differences varied among treatments and distance metrics. TSP1000 exhibited the largest difference from the control based on Bray–Curtis and weighted UniFrac distances, whereas TPA200 and TPA250 showed the strongest differences based on Jaccard and unweighted UniFrac distances. Exploratory within-formulation comparisons identified significant differences between all inclusion levels across all four metrics, except for TMC500 versus TMC1000 and TPA200 versus TPA250 using weighted UniFrac. Complete pairwise PERMANOVA results are provided in Supplementary Table S5.

#### 3.2.4. Taxonomic Composition of the Caecal Microbiota

**Figure 5:**
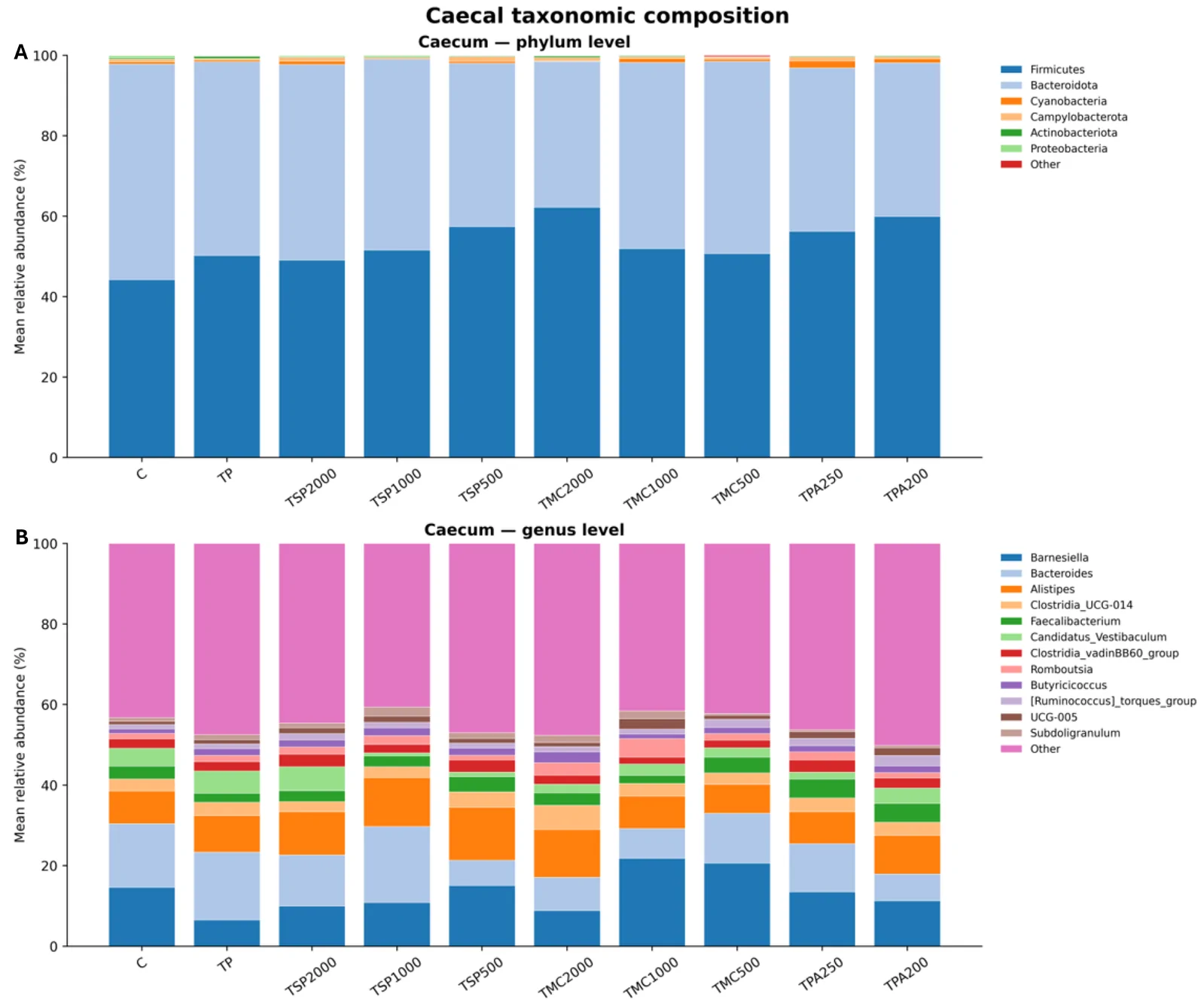
Taxonomic composition of the caecal microbiota at the (**A**) phylum and (**B**) genus levels. Bars show mean relative abundances within each dietary treatment group. Taxonomic labels such as Clostridia_UCG-014, Clostridia_vadinBB60_group, and UCG-005 represent provisional taxonomic designations retained from the SILVA database. “Other” includes taxa with low relative abundances.

The caecal microbiota was dominated by Firmicutes (53.1%) and Bacteroidota (45.0%), which together accounted for approximately 98.1% of all sequences. Minor phyla collectively represented less than 2% of the community. A total of 110 bacterial families and 260 genera were identified across all samples. The most abundant families were *Lachnospiraceae*, *Barnesiellaceae*, *Bacteroidaceae*, *Rikenellaceae*, *Oscillospiraceae* and *Ruminococcaceae*, whereas the dominant genera included *Barnesiella*, *Bacteroides*, *Alistipes, Faecalibacterium*, *Candidatus Vestibaculum*, *Romboutsia* and *Butyricicoccus* (Figure 5 and Supplementary Figure S3). Several of these taxa are associated with anaerobic fermentation and short-chain fatty acid production. Opportunistic pathogens, including the genera *Escherichia*, *Shigella* and *Campylobacter*, were detected only at very low relative abundances.

#### 3.2.5. Firmicutes, Bacteroidota and Firmicutes to Bacteroidota Ratio

Firmicutes and Bacteroidota were the dominant bacterial phyla across all treatment groups. The control group was characterized by a predominance of Bacteroidota (53.60 ± 4.43%) over Firmicutes (44.21 ± 4.68%), resulting in the lowest Firmicutes/Bacteroidota (F/B) ratio (0.83 ± 0.15). Several experimental groups exhibited a shift toward Firmicutes dominance, particularly TMC2000, TPA200 and TSP500, which showed the highest relative abundances of Firmicutes and the lowest abundances of Bacteroidota. Firmicutes abundance and the F/B ratio were significantly increased in TP, TSP500, TMC2000, TPA250 and TPA200 (Supplementary Figure S5).

#### 3.2.6. Family-Level Differential Abundance Analysis

Family-level ANCOM-BC2 analysis identified 35 bacterial families that differed significantly among treatment groups. The strongest global effects were observed for *Tannerellaceae*, *Muribaculaceae*, *Bacteroidaceae*, *Erysipelotrichaceae*, *Helicobacteraceae*, *Barnesiellaceae*, *Marinifilaceae*, *Butyricicoccaceae*, *Peptostreptococcaceae* and *Rikenellaceae*.

Compared with the control group, most treatments were characterized by reductions in *Muribaculaceae*, whereas decreases in *Bacteroidaceae* and *Tannerellaceae* and increases in *Erysipelotrichaceae*, *Marinifilaceae* and *Peptostreptococcaceae* were observed in specific treatment groups (Supplementary Figure S6). Pairwise comparisons demonstrated distinct treatment-specific responses, particularly among TSP500, TMC2000 and TMC1000, which exhibited the largest shifts in family composition.

#### 3.2.7. Differential Abundance Analysis

**Figure 6:**
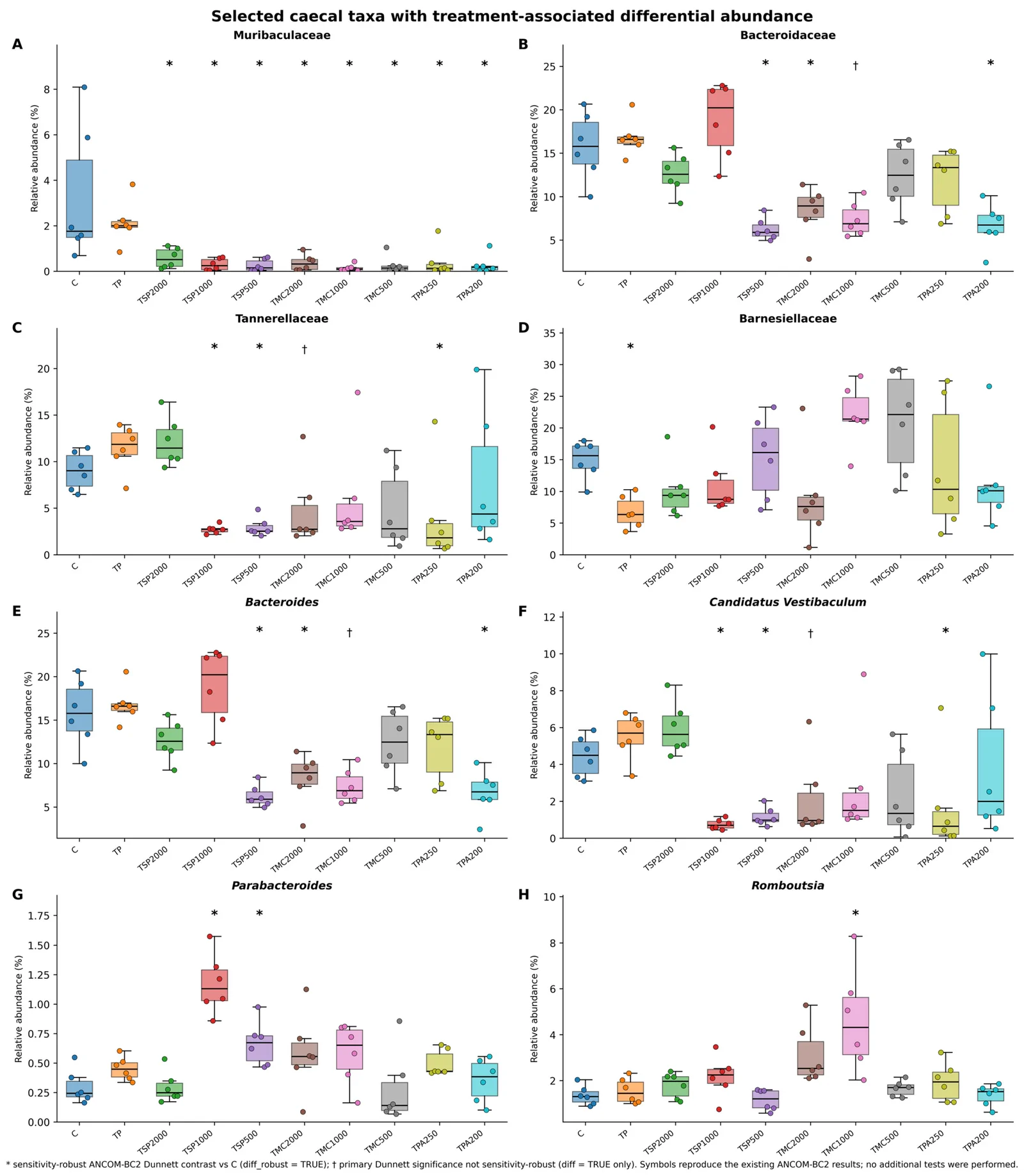
Relative abundances of selected caecal bacterial taxa across dietary treatment groups: (**A**) *Muribaculaceae*, (**B**) *Bacteroidaceae*, (**C**) *Tannerellaceae*, (**D**) *Barnesiellaceae*, (**E**) *Bacteroides*, (**F**) *Candidatus Vestibaculum*, (**G**) *Parabacteroides* and (**H**) *Romboutsia*. Points represent individual birds. Symbols indicate significant ANCOM-BC2 Dunnett comparisons with the control: * significant after multiple-testing correction and sensitivity analysis; † significant after multiple-testing correction but not supported by sensitivity analysis.

Genus-level ANCOM-BC2 analysis identified 82 taxa that differed significantly among treatment groups. The strongest global effects were observed for *Candidatus Vestibaculum*, *Subdoligranulum*, *Bacteroides*, *unclassified Barnesiellaceae* and genus *Parabacteroides*, *Helicobacter*, *Rikenella*, *Odoribacter*, *Butyricimonas*, *Negativibacillus*, *Barnesiella*, *Fournierella*, *Anaerostipes* and *Romboutsia*. Compared with the control group, several treatments were characterized by reductions in genus *Bacteroides* (TSP500, TMC2000 and TPA200), *Barnesiella* (TP) and *Candidatus Vestibaculum* (TSP1000, TSP500 and TPA250), whereas increases in genus *Parabacteroides* (TSP1000 and TSP500), *Odoribacter* (TSP1000), *Fournierella* (TSP2000), *Negativibacillus* (TSP500) and *Romboutsia* (TMC1000) were observed (Supplementary Figure S6). Selected treatment-associated differences are shown in Figure 6. Pairwise comparisons further demonstrated distinct treatment-specific responses, particularly involving TSP500, TMC500 and the TPA groups, which exhibited the greatest differences in genus composition. Complete family- and genus-level ANCOM-BC2 results are provided in Supplementary Table S6.

## 4. Discussion

The present study demonstrates that supplementation with different formulations and concentrations of sweet chestnut tannins (SCT) induced compartment-specific changes in the gut microbiota of broiler chickens, with more pronounced responses in the caecum than in the ileum. This finding is consistent with the distinct physiological and ecological characteristics of these intestinal segments. The ileum serves primarily as a site of nutrient digestion and absorption and is characterized by relatively rapid digesta transit, higher oxygen availability and lower microbial density, whereas the caeca harbor highly diverse anaerobic microbial communities specialized in the fermentation of undigested dietary substrates and the production of short-chain fatty acids (SCFAs) [4,22,23,24]. Although SCT supplementation significantly affected richness and phylogenetic diversity in the ileum, the absence of changes in Shannon diversity and Pielou’s evenness suggests that the dominant bacterial populations remained largely stable. In contrast, all four alpha-diversity metrics differed significantly among treatments in the caecum, accompanied by pronounced differences in community composition and a broader range of differentially abundant taxa. Daghio et al., however, did not find significant differences in caecal alpha or beta diversity after chestnut tannin supplementation, indicating that the effects of tannins on the microbiota may vary with formulation and inclusion level [16].

The taxonomic composition of the ileum remained typical of mature broiler chickens, being strongly dominated by members of the *Lactobacillaceae*, particularly the genera *Lactobacillus*, *Ligilactobacillus* and *Limosilactobacillus*, which are well adapted to the nutrient-rich conditions of the small intestine and play important roles in carbohydrate metabolism, competitive exclusion of pathogens and maintenance of intestinal homeostasis [4,25]. Despite significant differences detected by all beta-diversity metrics, differential abundance analyses identified only a small number of globally affected taxa and no significant treatment-specific differences relative to the control group. The stability of *Lactobacillaceae* across all treatments further suggests that SCT supplementation did not substantially disrupt the core ileal microbiota. Instead, treatment-related differences were mainly reflected in beta diversity, while significant global differences in taxonomic abundance were restricted to a small number of bacterial families, including *Aerococcaceae, Staphylococcaceae*, *Bacillaceae* and *Streptococcaceae*.

The significant differences observed across alpha- and beta-diversity metrics indicate that SCT supplementation affected the caecal microbial community beyond changes in individual taxa. Similar alterations in caecal diversity have been reported following dietary interventions in broiler chickens, reflecting the high responsiveness of the caecal microbiota to changes in feed composition and substrate availability [3,4]. Although previous studies have discussed tannin-associated microbial shifts in relation to gut health [10], the compositional changes observed here cannot establish improved intestinal health or exclude dysbiosis without supporting functional measurements.

The caecal microbiota of the control birds was characterized by the predominance of Firmicutes and Bacteroidota together with abundant members of the families *Lachnospiraceae*, *Ruminococcaceae*, *Oscillospiraceae* and *Bacteroidaceae*, a community structure typical of the mature broiler caecum and specialized for the fermentation of complex dietary substrates and SCFA production [4,26,27,28]. Several tannin treatments increased the relative abundance of Firmicutes and reduced Bacteroidota, resulting in higher Firmicutes/Bacteroidota ratios. Although higher Firmicutes abundance has been associated with beneficial intestinal effects in broilers [29], the functional significance of these changes remains uncertain because microbial metabolites and host responses were not assessed. In the present study, higher F/B ratios coincided with reductions in several dominant families within Bacteroidota, particularly *Muribaculaceae*, *Tannerellaceae* and *Bacteroidaceae*, suggesting that the phylum-level shifts reflected changes in several major components of the caecal bacterial community.

Several SCT formulations (particularly TSP1000, TSP500, TMC2000, TPA250 and TPA200) were associated with reductions in several dominant families within Bacteroidota, indicating that members of this phylum were among the bacterial groups most responsive to SCT supplementation. *Muribaculaceae* exhibited the most consistent response, with significantly lower abundance observed in nearly all experimental groups. Similar responsiveness of this lineage to dietary interventions has been reported previously in broilers [30,31]. Members of *Muribaculaceae* are increasingly recognized as specialized degraders of complex polysaccharides and contributors to SCFA production, although most mechanistic evidence originates from non-avian systems [32]. The biological significance of the observed reduction in *Muribaculaceae* remains unclear. Nevertheless, the consistent response across different tannin formulations and concentrations suggests that *Muribaculaceae* may be particularly susceptible to the SCT-induced effects.

*Bacteroidaceae* and *Tannerellaceae* also exhibited significant reductions in selected treatment groups, including TSP500, TMC2000 and TPA200 for *Bacteroidaceae*, and TSP1000, TSP500 and TPA250 for *Tannerellaceae*. Members of these families are common constituents of the mature chicken caecum and contribute substantially to the degradation of complex dietary polysaccharides and subsequent fermentation processes [4,33]. At the genus level, these changes were accompanied by significantly lower abundance of *Bacteroides* in TSP500, TMC2000 and TPA200. Members of the genus *Bacteroides* are highly adapted to distal-gut carbohydrate degradation and have been associated with enriched polysaccharide degradation pathways, increased caecal SCFA concentrations and intestinal homeostasis in broilers [34,35]. However, reductions in *Bacteroides* should not necessarily be considered detrimental, as previous studies have reported decreases in this genus alongside increases in other fermentative bacteria, including members of *Lachnospiraceae* and *Ruminococcaceae* [10,15].

Reductions in several dominant bacterial families were accompanied by increases in other anaerobic genera, indicating that the response to SCT differed among caecal bacterial taxa. In particular, genus *Parabacteroides* increased in TSP1000 and TSP500, whereas genus *Odoribacter* increased in TSP1000. The genera *Fournierella* and *Romboutsia* were enriched in TSP2000 and TMC1000, respectively. Because the caecum represents the principal site of microbial fermentation in chickens, these shifts are most relevant through their potential effects on fermentation processes, SCFA production and intestinal homeostasis. *Parabacteroides* spp. and *Odoribacter* spp. are strict anaerobes associated with complex carbohydrate utilization and SCFA metabolism [4,36]. Species of the genus *Parabacteroides* have been linked to intestinal development and improved feed efficiency in broilers, and *Parabacteroides distasonis* has recently been incorporated into probiotic formulations developed for poultry production [37]. Likewise, *Odoribacter* spp. have emerged as a potential next-generation probiotic candidate because of their associations with intestinal health and host metabolic regulation [36]. Although the functional consequences of their enrichment cannot be determined without direct metabolomic measurements, the increase in these genera suggests that specific SCT formulations may preserve or redirect fermentative capacity toward alternative anaerobic populations following reductions in other members of Bacteroidota. The increase in *Fournierella* in TSP2000 is of interest because greater abundance of this genus has previously been associated with improved feed efficiency in chickens [38]. Similarly, *Romboutsia*, which increased in TMC1000, is a metabolically versatile anaerobe capable of utilizing a range of carbohydrates through fermentative metabolism [39]. The functional roles discussed above for the genera *Parabacteroides*, *Odoribacter*, *Fournierella* and *Romboutsia* are based on previous studies. Their increased abundance in this study does not establish increased microbial activity or benefits to the host, as these were not directly assessed.

Several additional bacterial taxa exhibited treatment-specific responses to SCT supplementation. *Erysipelotrichaceae* was enriched exclusively in TSP500, whereas no significant increase was observed in TSP1000 or TSP2000. Members of this family have previously been associated with feed efficiency and body weight gain in poultry, although their ecological role and functional significance remain incompletely understood [4]. The increase observed only in TSP500 indicates that the response of *Erysipelotrichaceae* did not follow a simple dose-dependent pattern. In TMC1000, the increase in *Peptostreptococcaceae* was accompanied by enrichment of *Romboutsia*, a genus within this family, indicating a consistent response at both family and genus levels. *Barnesiellaceae* abundance was reduced only in the TP group. Although members of this family have been associated with SCFA concentrations and growth-related host–microbiota interactions in poultry [40,41,42], the biological significance of this isolated response remains uncertain.

The observed responses varied among treatments, but the incomplete factorial design does not allow formulation and concentration effects to be fully separated. Differences in significance relative to the control do not establish differences between doses, and dose–response patterns require exploratory comparisons within each formulation. Similar observations have been reported in previous poultry studies, where different tannin products generated divergent effects on bacterial diversity and taxonomic composition despite comparable inclusion levels [10,43]. Protein complexation and microencapsulation can influence the stability, gastrointestinal availability and release profile of phytogenic compounds, potentially altering the timing and extent of microbial exposure [7,11,12], which may partly explain the different microbial responses observed among the SCT treatments.

Among the tested treatments, TSP1000 and TMC1000 showed some of the most interesting changes in caecal microbiota. TSP1000 increased the abundance of *Parabacteroides* and *Odoribacter*, genera associated with carbohydrate fermentation and SCFA metabolism, whereas TMC1000 increased *Romboutsia* and *Peptostreptococcaceae*. TSP2000 was also of interest because of the increase in *Fournierella*, a genus previously associated with improved feed efficiency in chickens [38]. In contrast, TSP500 affected a larger number of bacterial taxa, but these changes cannot necessarily be considered beneficial. Based on their enrichment of taxa previously associated with carbohydrate fermentation and SCFA metabolism, TSP1000 and TMC1000 may be candidates for further studies measuring microbial metabolites, intestinal health and growth performance, which were not assessed here, as microbial changes alone do not establish health benefits.

Previous studies have described several mechanisms through which tannins may influence gut microbiota, including antimicrobial activity [12], binding to proteins and other macromolecules [11,12], and bacterial conversion into smaller compounds with altered biological activity [11]. Chestnut tannins have also been reported to influence intestinal barrier function and inflammatory responses in heat-stressed broilers [14]. These mechanisms could contribute to the microbial changes observed here, but none was directly assessed in the present experiment, so their involvement remains hypothetical.

Several limitations should be considered when interpreting the present findings. The study relied on 16S rRNA gene sequencing, which provides information on microbial composition but does not directly assess microbial function. Microbial metabolites, host physiological responses and bird growth performance were not measured, limiting our ability to determine the biological significance of the observed changes. Controls containing the formulation carriers without tannins were not included. Therefore, the observed effects apply to the complete formulations and cannot be attributed solely to tannins. The incomplete factorial design and limited product characterization restrict the separation of formulation and concentration effects and their attribution to specific product properties. Another limitation is the small sample size per treatment, which may have limited statistical power to detect smaller treatment effects and warrants caution when interpreting treatment-specific differential abundance. Sampling only on day 42 precludes assessment of changes over time, while different bioinformatic workflows limit direct comparisons between intestinal compartments. In addition, the mechanisms responsible for the different responses among SCT formulations remain unclear and should be addressed in future studies integrating multi-omics analyses.

## 5. Conclusions

Dietary supplementation with SCT induced significant, compartment-specific changes in the gut microbiota of broiler chickens. The ileal microbiota remained largely stable, with *Lactobacillaceae* remaining the dominant bacterial family despite significant differences in overall community composition. In contrast, the caecal microbiota showed more pronounced changes, including differences in alpha and beta diversity, and responses across numerous bacterial families and genera. An increase in the relative abundance of Firmicutes was observed, while the proportion of Bacteroidota simultaneously decreased. These responses varied among SCT formulations and concentrations and did not follow a consistent dose–response pattern. The results indicate that the effects of SCT on the broiler gut microbiota depend on both the intestinal compartment and the form and concentration in which the tannins are administered, with the caecum showing the strongest microbial response. The implications of these microbial changes for bird health and performance require further investigation.

## Figures and Tables

**Table 1:** Dietary treatments applied to broiler chickens.

Group	Sweet Chestnut Tannin Formulation	Sweet Chestnut Tannin Concentration (mg/kg Feed)
C	No tannin supplementation	–
TP	Powdered tannins	1000
TSP_2000_	Soy protein-bound tannins	2000
TSP_1000_	Soy protein-bound tannins	1000
TSP_500_	Soy protein-bound tannins	500
TMC_2000_	Microencapsulated tannins	2000
TMC_1000_	Microencapsulated tannins	1000
TMC_500_	Microencapsulated tannins	500
TPA_250_	Orthophosphoric acid-bound tannins	250
TPA_200_	Orthophosphoric acid-bound tannins	200

**Table 2:** Composition of the sweet chestnut tannin formulations.

Characteristic	TP	TSP	TMC	TPA
Sweet chestnut extract (%)	100	50	50	80
Other components (%)	None	Soybean protein, 50	Palm oil, 50	Orthophosphoric acid, 20
Formulation	Powdered extract	Soybean protein-bound extract	Microencapsulated extract	Acid-hydrolysed extract
Calculated tannin content (%)	75	37.5	37.5	60

**Table 3:** Ingredient and calculated nutrient composition of the basal diets.

Item	Starter	Grower	Finisher
Feeding period (days)	1–14	15–28	29–42
Ingredient composition (%)			
Maize	41.612	42.292	43.520
Soybean cake	32.134	28.533	26.052
Wheat	12.500	12.500	12.500
Wheat feed flour	4.000	5.000	5.000
Sunflower meal	4.000	5.000	5.000
Soybean oil	0.125	1.196	2.679
Monocalcium phosphate	0.629	0.479	0.249
Feed supplement ^1^	5.000	5.000	5.000
Total	100.000	100.000	100.000
Calculated nutrient composition (g/kg)			
Crude protein	210.00	200.00	190.00
Crude fat	29.69	38.43	51.56
Crude fibre	45.17	46.20	45.17
Crude ash	40.87	38.48	34.59
Calcium	9.60	8.70	7.90
Available phosphorus	4.80	4.50	4.00
Digestible lysine	12.80	11.50	10.30

^1^ Included a feed flour carrier, limestone, amino acids, salt, sodium bicarbonate, choline, vitamins, trace minerals, enzymes and antioxidant; betaine was additionally included in the finisher diet.

## Data Availability

The raw sequencing data generated in this study have been deposited in the NCBI Sequence Read Archive (SRA) under BioProject accession number PRJNA1551761. Additional data are provided in the article and Supplementary Materials.

## Supplementary Materials

The following supporting information can be downloaded at https://www.mdpi.com/article/10.3390/ani16213514/s1: Figure S1: Ileal alpha diversity across dietary treatment groups; Figure S2: Principal coordinate analysis of ileal and caecal microbiota based on Jaccard and unweighted UniFrac distances; Figure S3: Family-level taxonomic composition of the ileal and caecal microbiota; Figure S4: Observed amplicon sequence variant rarefaction curves for ileal and caecal samples; Figure S5: Relative abundances of Firmicutes and Bacteroidota and the Firmicutes/Bacteroidota ratio in caecal samples; Figure S6: Heatmaps of family- and genus-level ANCOM-BC2 differential abundance results for caecal microbiota relative to the control group; Table S1: Sequencing and Deblur processing statistics for ileal samples; Table S2: Pairwise PERMANOVA results for ileal microbiota; Table S3: Family- and genus-level ANCOM-BC2 results for ileal microbiota; Table S4: Sequencing and DADA2 processing statistics for caecal samples; Table S5: Pairwise PERMANOVA results for caecal microbiota; Table S6: Family- and genus-level ANCOM-BC2 results for caecal microbiota.

## Abbreviations

The following abbreviations are used in this manuscript:

SCT	Sweet chestnut tannins
SCFAs	Short-chain fatty acids
ASVs	Amplicon sequence variants
PCoA	Principal coordinate analysis
PERMANOVA	Permutational multivariate analysis of variance
ANCOM-BC2	Analysis of compositions of microbiomes with bias correction 2
F/B	Firmicutes/Bacteroidota ratio
FDR	False discovery rate
C	Unsupplemented control
TP	Powdered tannins
TSP	Soy protein-bound tannins
TMC	Microencapsulated tannins
TPA	Orthophosphoric acid-bound tannins
